# Monocyte‐derived dendritic cells enhance protection against secondary influenza challenge by controlling the switch in CD8^+^ T‐cell immunodominance

**DOI:** 10.1002/eji.201646523

**Published:** 2016-12-09

**Authors:** Jazmina L. G. Cruz, José V. Pérez‐Girón, Anja Lüdtke, Sergio Gómez‐Medina, Paula Ruibal, Juliana Idoyaga, César Muñoz‐Fontela

**Affiliations:** ^1^ Heinrich Pette InstituteLeibniz Institute for Experimental VirologyHamburgGermany; ^2^ Bernhard Nocht Institute for Tropical MedicineHamburgGermany; ^3^ German Center for Infection Research (DZIF)Partner Site HamburgHamburgGermany; ^4^ Department of Microbiology and ImmunologyStanford University School of MedicineStanfordCAUSA

**Keywords:** CD8^+^ T cell · Dendritic cell · Immunodominance · Influenza virus · Monocyte

## Abstract

Influenza virus infection triggers an increase in the number of monocyte‐derived dendritic cells (moDCs) in the respiratory tract, but the role of these cells during antiviral immunity is still unclear. Here we show that during influenza infection, moDCs dominate the late activation of CD8^+^ T cells and trigger the switch in immunodominance of the CD8^+^ T‐cell response from acidic polymerase specificity to nucleoprotein specificity. Abrogation of monocyte recruitment or depletion of moDCs strongly compromised host resistance to secondary influenza challenge. These findings underscore a novel function of moDCs in the antiviral response to influenza virus, and have important implications for vaccine design.

## Introduction

Influenza is a highly contagious, acute, febrile, respiratory disease, which causes seasonal epidemics and, at random intervals, pandemics with significant morbidity and mortality 1. Antigen‐specific CD8^+^ T cells play a crucial role in viral clearance through their ability to eliminate influenza‐infected cells during both primary and secondary infections 2. In the case of the C57BL/6 mouse model of infection, CD8^+^ T cells are mainly directed to a relatively few immunodominant peptides 3. CD8^+^ T cells specific for the acidic polymerase (PA)_224‐233_ epitope dominate early effector responses, while nucleoprotein (NP)_366‐374_‐specific T cells achieve a much greater magnitude during secondary challenge, constituting between 80–90% of all influenza‐specific CD8^+^ T cells 4. This sequential formation of virus‐specific CD8^+^ T cells has been attributed to differences in antigen presentation patterns. Thus, while only “classic” dendritic cells seem capable of priming PA‐specific CD8^+^ T cells, NP‐specific T‐cell immunity can be driven by both classic DCs as well as other undefined populations which notably, expressed high levels of CD11b 5. Previous studies have further investigated the nature of the DCs responsible for the switch between PA‐ to NP‐specific CD8^+^ T‐cell responses suggesting a division of labor. On one hand, migratory CD11b^+^ DCs were proposed to be responsible to trigger this immunodominance switch through late antigen presentation during primary infection, but the specific DC subset involved was not determined 6. On the other hand, CD103^+^ DCs were shown to be required for early proliferation of NP‐specific T cells after reinfection 7. Thus, while it is clear that the immunodominance switch from PA‐ to NP‐specific CD8^+^ T cells is crucial for the generation of protective influenza‐specific T‐cell memory, the physiological mechanism responsible for it, as well as its timing and location is not fully understood. The identification of the DC subsets responsible for mediating this immunodominance switch is crucial for the development of DC‐targeted vaccines 8, 9.

The DC lineage can be classified on the basis of phenotype and origin. CD103^+^ DCs are lung‐resident cells that differentiate from bone marrow‐derived pre‐DCs 10, 11, and are defined by their dependence on the transcription factors *Irf8*, *Batf3*, and *Id2*, as well as the cytokine receptor Flt3 12. Early after influenza infection, CD103^+^ DCs capture virus particles, migrate efficiently to the lung‐draining mediastinal lymph nodes (mLNs), where they dominate cross‐priming of (PA)_224‐233_‐specific CD8^+^ T cells 13, 14, 15. Later during influenza infection, CD11b‐expressing cells can prime CD8^+^ T cells 16, however, the role of this late antigen presentation in the context of host antiviral responses is poorly understood.

Activated monocytes are CD11b^+^ myeloid circulating cells that infiltrate the lungs of influenza‐infected mice in a CCR2‐dependent manner and vastly outnumber any resident DC population 17, 18. The plasticity of monocytes to differentiate into monocyte‐derived DCs (moDCs) and macrophages, and their high number under inflammatory conditions suggests an important role in the antiviral response, but the specific functions of monocytes during influenza immunity in vivo are incompletely defined. Addressing the specific role of moDCs on antiviral immunity has been a difficult task so far, partially due to the lack of strategies to differentiate these cells from other myeloid populations, such as macrophages, immature monocytes, and resident CD11b^+^ DCs 19. Recently, the ImmGen Project as well as other studies have provided valuable clues about the phenotype of moDCs, including the conserved expression of Ly6C, CD64, Mar‐1, and CD209, i.e. the mouse equivalent to human DC‐SIGN 20, 21, 22, 23. In this study, we have taken advantage of the Ly6C phenotypic marker to distinguish moDCs from resident CD11b^+^ DCs during infection, and to elucidate the physiological role of moDCs during influenza‐specific CD8^+^ T‐cell immunity. We found that, during the late phase of the primary response, moDCs were the main antigen‐presenting cell population and triggered the immunodominance switch from PA‐ to NP‐specific CD8^+^ T cells. Importantly, specific blockade of monocyte recruitment or targeted depletion of CD11b^+^ cells reduced the formation of virus‐specific memory CD8^+^ T cells and compromised protection against secondary influenza challenge. Our findings highlight the protective role of moDCs on influenza virus infection, and warrant their consideration as targets for rational design of future influenza vaccines.

## Results and discussion

### moDCs infiltrate the murine lung shortly after H1N1 infection

**Figure 1:**
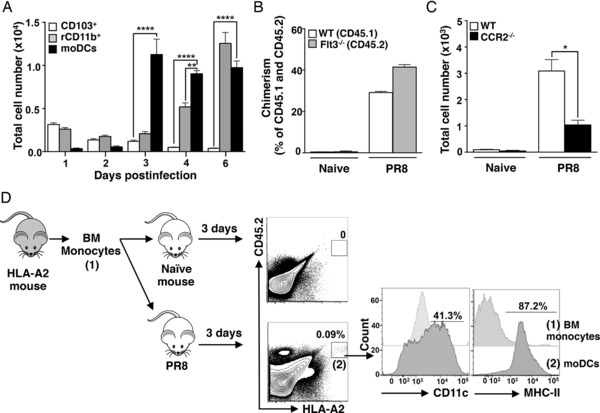
Influenza infection promotes recruitment of monocytes and triggers their differentiation into moDCs. (A) WT mice were infected intranasally with 250 PFU of PR8 and absolute numbers of CD103^+^ DCs, lung resident CD11b^+^ DCs (rCD11b) and moDCs were determined at the indicated time points (*n* = 3 mice per time point) in the lungs by flow cytometry. (B) WT/Flt3^−/−^ mixed BM chimeric mice were infected intranasally with 250 PFU of PR8 and the frequency of moDCs was evaluated in the lungs 4 dpi by flow cytometry. The frequency of moDCs in the CD45.1 (WT) or CD45.2 (Flt3^−/−^) gates is shown. Data are shown as mean ± standard error of the mean of *n* = 4 mice. (C) WT and CCR2^−/−^ mice were infected intranasally with 250 PFU of PR8. Absolute cell number of moDCs in the singlet population was determined at 4 dpi in the lungs. Data are shown as mean ± SEM of *n* = 4 mice. (A–C) Graphs depict one representative experiment of at least three experiments. (D) Monocytes were sorted as SSC‐A^low^ CD11c^−^ MHC‐II^−^ CD11b^+^ Ly6C^+^ cells from the BM of donor HLA‐A2^+^ transgenic mice. Purified monocytes were injected in naïve mice or in mice infected with PR8 for 3 days. Twenty‐four hours later, the phenotype of donor cells (0.09% of the singlet population and 3% of the CD11c^+^ MHC class II^hi^ cell population) was determined in the lungs by flow cytometry. Data shown are from a single experiment performed with *n* = 5 mice. Two independent experiments were performed. In all cases, data are shown as mean ± SEM. Asterisks represent statistical significance as follows: *p < 0.05; **p < 0.005; ****p < 0.0001 as assessed by one‐way ANOVA followed by Bonferroni's posttest.

Murine lung DCs have been identified so far by flow cytometry as CD11c^+^ MHC class II^hi^ Siglec‐F^−^ cells 24, 25. However, this strategy does not allow discrimination between resident CD11b^+^ DCs and moDCs. In order to specifically dissect the functions of moDCs during influenza infection of mice, we sought to establish a flow cytometry panel that allowed us to distinguish them from other myeloid cells expressing CD11b. Since infiltrating moDCs in the dermis, gut mucosa, and lymphoid tissues retain monocyte markers such as Ly6C and CD209 21, 22, 26, the use of these markers to track lung moDCs after infection was evaluated. Shortly after intranasal infection with the mouse‐adapted A/Puerto Rico/8/34 (H1N1) influenza virus (hereafter referred to as PR8), we observed a population consistent with moDCs, which expressed CD11b^+^, but not CD103^+^, and showed high expression of Ly6C^+^ (Supporting Information Fig. 1). This population also showed co‐expression of CD64 and Mar‐1, characteristic of moDCs 23 (Supporting Information Fig. 2). The number of these cells in the lung peaked at day 3 postinfection in coincidence with the peak of virus replication (Fig. 1A and data not shown).

Monocytes and DCs arise from common monocyte‐DC precursors in the BM, but separate early during hematopoiesis in two different lineages: Flt3‐Flt3L‐dependent pre‐DCs and common monocyte progenitors, respectively 27. To determine whether our identified inflammatory leukocyte population was dependent on Flt3 signaling, mixed BM chimeric mice were engineered by transplantation of 50% WT and 50% Flt3^−/−^ BM into lethally irradiated WT mice. After PR8 infection, loss of Flt3 signaling did not affect the accumulation of CD11b^+^ Ly6C^hi^ cells indicating that these cells were not classic DCs (Fig. 1B). On the other hand, CD11b^+^ Ly6C^hi^ cells were significantly reduced in PR8‐infected CCR2^−/−^ mice, supporting their monocytic origin 17, 28 (Fig. 1C). To further confirm that CD11b^+^ Ly6C^hi^ cells were in fact moDCs, FACS‐purified BM monocytes from HLA‐A2^+^ transgenic donor mice were transferred into WT recipient mice after PR8 infection. The presence of surface HLA‐A2 in donor cells allowed us to track their fate upon infection. Our results indicated that these transferred monocytes infiltrated only the lungs of infected mice, where they upregulated CD11c and MHC class II (Fig. 1D). Taken together, our findings demonstrate that under nflammatory conditions, blood‐borne monocytes acquire APC features while recruited to the lung in a CCR2‐dependent manner, and that these cells can be distinguished from lung‐resident CD11b^+^ DCs based on the expression of Ly6C, Mar‐1, and CD64.

### Depletion of moDCs reduces protection against secondary influenza infection

**Figure 2:**
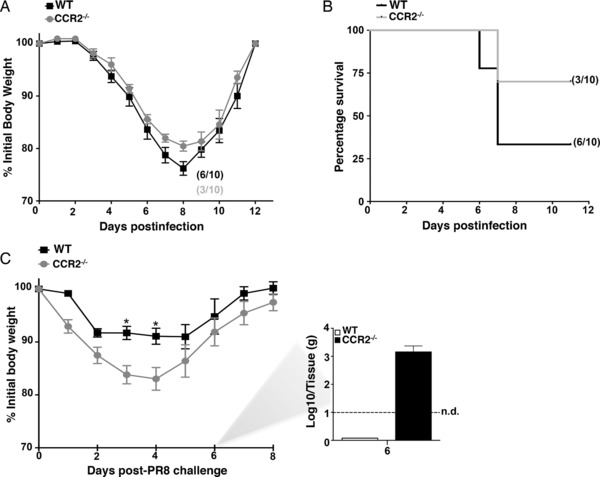
MoDCs confer protection against secondary influenza challenge. (A) WT and CCR2^−/−^ mice (*n* = 10) were infected intranasally with 500 PFU of PR8 (LD_50_). Animals were monitored daily for weight loss and clinical signs for up to 12 days. (B) Kaplan–Meier survival curve showing the percentage of mice that survived infection. (C) WT and CCR2^−/−^ animals (*n* = 10) were infected intranasally with 500 PFU of x‐31 virus. Twenty‐one days after infection mice were challenged with a lethal dose of PR8 (10^4^ PFU). Animals were monitored daily for weight loss and clinical signs for up to 10 days (left panel). Viral titers in the lungs of PR8‐infected mice at 6 dpi were determined by plaque assay (right). (A–C) Results represent *n* = 10 mice pooled from three independent experiments. Throughout the figure data are shown as mean ± SEM. Asterisks represent statistical significance (*p < 0.05) as assessed by one‐way ANOVA followed by Bonferroni's posttest.

To gain insight into the physiological role of infiltrating moDCs during primary influenza infection, we took advantage of the CCR2^−/−^ mouse model in which infiltration of activated monocytes into inflamed peripheral tissues is reduced up to 95% (Fig. 1C). WT and CCR2^−/−^ mice were infected with a lethal dose of PR8 and their morbidity (weight loss) and mortality was compared. Although both groups lost weight comparably after infection (Fig. 2A), the lack of monocyte infiltration in CCR2^−/−^ mice resulted in enhanced survival (Fig. 2B). These results are in agreement with the previously described role of inflammatory monocytes on influenza immunopathology 29.

To address the effect of monocyte infiltration in secondary influenza challenge, we used a recall model based on primary infection with x‐31 influenza virus (H3N2) and secondary infection with PR8 virus (H1N1) 21 days later (Fig. 2C). Since PR8 and x‐31 share the same internal proteins but have different virion surface proteins, this system allows the evaluation of secondary antigen‐specific CD8^+^ T‐cell responses without the influence of protective antibodies against the virus hemagglutinin 30. While the number of mice surviving lethal challenge were comparable between both groups (eight out of nine mice), CCR2^−/−^ mice lost significantly more weight than WT mice indicating enhanced virus‐associated morbidity (Fig. 2C). Consistently, WT mice were able to clear virus from the lungs by day 6 postinfection, a time point in which 10^3^ plaque‐forming units in CCR2^−/−^ mice could be still detected (Fig. 2C, right panel). These results indicated that loss of moDC function reduced host resistance to secondary influenza virus infection, and raised questions regarding the physiological mechanism governing this effect.

### MoDCs modulate the immunodominance switch of virus‐specific CD8^+^ T cells

**Figure 3:**
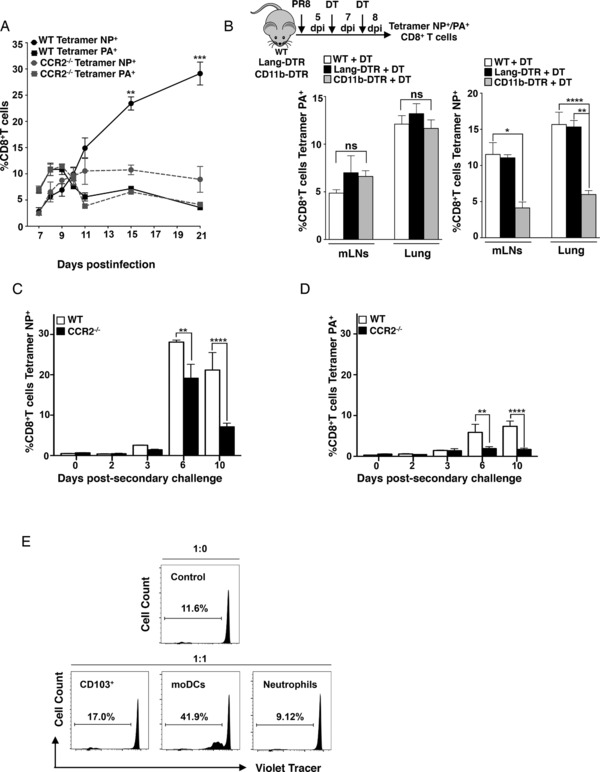
MoDCs drive the switch in immunodominance of the influenza‐specific CD8^+^ T‐cell response. (A) WT and CCR2^−/−^ mice were intranasally infected with 250 PFU of PR8. The frequency of NP_366‐374_ and PA_224‐233_‐specific CD8^+^ T cells was determined in the lungs of infected mice at 7, 8, 9, 10, 11, 15, and 21 dpi by dextramer staining and flow cytometry (*n* = 5—8 per time point). (B) WT, Langerin‐DTR, and CD11b‐DTR mice (*n* = 6) were intranasally infected with 250 PFU of PR8. At day 5 postinfection, mice were treated with 0.5 μg of DT by intraperitoneal injection. A second dose of DT was administered at day 7 postinfection. At day 8 postinfection, lungs and mLNs were harvested and the frequency ratio NP_366‐374_/PA_224‐233_‐specific CD8^+^ T cells was determined. (C and D) WT and CCR2^−/−^ (*n* = 15) animals were infected intranasally with 500 PFU of x‐31. Thirty days after infection mice were challenged with a lethal dose of PR8 (10^4^ PFU). Lungs and mLNs were collected at the indicated time points (*n* = 3 per time point) and the percentage of (C) NP_366‐374_ and (D) PA_224‐233_‐specific CD8^+^ T cells was determined. (A–D) Data are shown as mean ± SEM. Statistical significance is represented as follows: **p < 0.005; ***p < 0.0005 as assessed by one‐way ANOVA followed by Bonferroni's posttest. Data are representative experiments of at least three independent experiments. (E) Mice were intranasally infected with 250 PFU of PR8 and Neutrophils (MHC‐II^−^GR‐1^+^CD11b^+^, negative control), CD103 DCs (MHC‐II^+^CD11c^+^CD103^+^SSC^low^, positive control) and moDCs (MHC‐II^+^CD11c^+^CD11b^+^Ly6C^+^SSC^low^) were sorted from the mLNs of donor mice at day 7 after influenza virus infection. Antigen‐experienced T cells (CD3^+^CD8^+^CD44^+^) isolated from the mLNs of influenza‐infected mice were labeled with Cell Trace^TM^ Violet and cocultured for 72 h with the indicated APCs and ratios. Histograms show data from a single experiment representative of two independent experiments.

The switch from PA‐ to NP‐specific CD8^+^ T‐cell responses is required for proper establishment of virus‐specific T‐cell memory, and previous studies have suggested that cells expressing CD11b may play an important role on the modulation of the immunodominance hierarchies 5, 16. Thus, we hypothesized that moDCs could modulate this change in immunodominance via late antigen presentation 4, 6. To test our hypothesis, the accumulation of PA‐ and NP‐specific CD8^+^ T cells in infected lungs was compared between WT and CCR2^−/−^ mice. We observed that the switch from PA‐ to NP‐specific CD8^+^ T cells in WT mice occurred in the lung around days 9–10 postinfection (Fig. 3A). After this time point, NP‐specific CD8^+^ T cells dominated the influenza‐specific response and accumulated in the lungs of infected animals (Fig. 3A). Strikingly, CCR2^−/−^ mice showed an important impairment in their ability to switch from PA‐ to NP‐specific CD8^+^ T‐cell immunity, which resulted in significant reduction of NP‐specific CD8^+^ T cells in the lung (Fig. 3A). To rule out effects associated to the mouse model, we also evaluated the immunodominance switch in Langerin‐DTR and CD11b‐DTR mice, in which treatment with diphtheria toxin (DT) allowed specific deletion of CD103^+^ DCs (langerin^+^) and CD11b^+^ cells (Supporting Information Fig. 3). Mice were infected with PR8, treated with DT at days 5 and 7 postinfection, and the frequencies of NP‐ and PA‐specific CD8^+^ T cells were determined in mLNs and lungs at day 8 postinfection. In agreement with the findings in the CCR2^−/−^ model, depletion of CD11b^+^ cells, but not langerin^+^ cells, resulted in significant reduction of NP‐specific CD8^+^ T cells in the lung and mLNs (Fig. 3B), but did not influence the formation of PA‐specific CD8^+^ T cells.

We reasoned that the influence of moDCs in the formation of NP‐specific CD8^+^ T cells might result in enhanced formation of memory T cells and improved T‐cell responses to recall influenza virus challenge. To test this hypothesis, we infected WT and CCR2^−/−^ mice with x‐31 virus and 30 days later we subjected them to secondary infection with PR8 virus after which we evaluated NP‐ and PA‐specific CD8^+^ T‐cell formation over time. NP‐specific CD8^+^ T cells peaked at day 6 postsecondary challenge and were significantly higher in WT compared to CCR2^−/−^ mice at days 6 and 10 (Fig. 3C and Supporting Information Fig. 4). Moreover, WT mice also harbored significantly higher levels of PA‐specific CD8^+^ T cells albeit at lower levels (Fig. 3D). These findings strengthened the notion that the role of moDCs on late formation of NP‐specific T cells after primary influenza response resulted in improved responses to recall infection providing a rationale for the enhanced morbidity and virus titers observed in CCR2^−/−^ mice during secondary influenza infection (Fig. 2C).

Our results pointed out at moDCs as chief modulators of late CD8^+^ T‐cell priming but did not address whether this effect was mediated by direct antigen presentation by moDCs 31, 32 rather than, for example, antigen transfer to other DCs 33, 34. Thus, we FACS‐purified moDCs (SSC‐A^low^ CD11c^hi^CD11b^hi^ Ly6C^hi^)cells, CD103^+^ DCs and neutrophils from the mLNs of PR8‐infected mice 7 days postinfection and incubated these cells with virus‐specific, antigen‐experienced (CD44^+^) polyclonal CD8^+^ T cells (Fig. 3E). Sorted CD8^+^ T cells alone or cocultured with neutrophils exhibited a basal level of homeostatic proliferation likely as a consequence of the T cell–DC coculture 35. Interestingly, at this time point, only moDCs cells were able to induce CD8^+^ T‐cell proliferation above this basal level (Fig. 3E). These results were consistent with a direct role of moDCs on late presentation of influenza antigen, and strongly suggested that the ability of moDCs to modulate the immunodominance switch was due to their capacity to induce antigen‐specific CD8^+^ T‐cell proliferation.

Our findings demonstrate that moDCs are required for the switch in influenza‐specific CD8^+^ T‐cell immunodominance, and that loss of moDC function prevents accumulation of NP‐specific T cells in the lungs which, in turn, compromised secondary immune responses to influenza virus. These results warrant consideration of moDCs as putative targets for DC‐based influenza vaccines.

### Concluding remarks

MoDCs triggered the immunodominance switch from PA‐ to NP‐specific CD8^+^ T‐cell responses. MoDCs modulate CD8^+^ T‐cell immunodominance switch via late presentation of virus antigen. Depletion of moDCs compromises secondary immune responses to influenza virus.

## Materials and methods

### Experimental influenza virus infection in mice

This study was carried out in strict accordance with the recommendations of the German Society for Laboratory Animal Science and under the supervision of a veterinarian. The protocol was approved by the Committee on the Ethics of Animal Experiments of the City of Hamburg (permit no. 104/12). All efforts were made to minimize the number of animals used for experiments and to alleviate suffering of animals during experimental procedures. All staff carrying out animal experiments passed an education and training program according to category B or C of the Federation of European Laboratory Animal Science Associations.

### Mice, reagents, and viruses

C57BL/6J, BALB/c, CD45.1^+^ congenic B6 mice, CCR2^−/−^ (B6.129S4‐Ccr2^tm1Ifc/J^), Langerin‐DTR (B6.129S2‐*Cd207tm3Mal*/J), CD11b‐DTR (FVB‐Tg(ITGAM‐DTR/EGFP)_34_Lan/J), and HLA‐A2.1 (C57BL/6Tg(HLA‐A2.1)1Enge/J) mice were purchased from Jackson Laboratories and bred at the Heinrich Pette Institut animal facility. Flt3^−/−^ mice were a kind gift from Claudia Waskow (Center for Regenerative Therapies, TU Dresden). Viral challenge was performed by inoculation of the virus solution in PBS directly to the nostrils of mice anesthetized with inhaled isoflurane. Influenza A/PR8/34 (H1N1) and x‐31 (H3N2) viruses were propagated in 10‐day‐old embryonated chicken eggs at 37°C.

### Cell preparation, flow cytometry, and cell sorting

In all experiments lungs were perfused with 5 mL of sterile PBS. Single cell suspensions were obtained from lungs and mLNs as previously described 36. Briefly, lungs and mLNs of infected animals were harvested and digested with Collagenase D (2 mg/mL, Roche) and DNAseI (50 μg/mL, Sigma). Single cell suspensions were treated with BD Pharm Lysing Buffer (BD Bioscience); cells were blocked with CD16/CD32 Fc‐Block antibody followed by staining with an antibody cocktail. All antibodies were purchased from BioLegend unless otherwise stated. Isotype controls and fluorescence minus‐one (FMO) controls were utilized for antibody panel setup. An FACScanto II or LSR Fortessa instrument (BD Biosciences) was used for flow cytometry. Staining of influenza‐specific CD8^+^ T cells was achieved by staining of H‐2^b^‐restricted NP_366‐374_‐specific or PA_224‐233_‐specific CD8^+^ T cells using commercial dextramers (Immudex). Analysis of data was performed with FlowJo software (Treestar). Mice were intranasally infected with 250 PFU of PR8 and the mLNs were collected, processed, and stained as described above. Antigen‐experienced T cells (CD3^+^CD44^+^CD8^+^B220^−^), CD103^+^ DCs (CD11c^+^ MHC‐II^hi^ SSC^low^ CD103^+^), CD11b^+^ cells (CD11c^+^ MHC‐II^hi^ SSC^low^ CD11b^+^), and neutrophils (Ly6G^+^ CD11b^+^) were sorted using high purity mode in an FACSAria I (BD Biosciences). T cells were labeled with Cell Trace Violet (Invitrogen). Briefly, 1 × 10^6^ cells/mL DMEM were incubated with 5 μM of Cell Trace Violet for 30 min at 37°C. The reaction was stopped by adding an equal volume of FCS. A total of 1 × 10^4^ Cell Trace Violet ‐labeled T cells were cocultured for 72 h with the indicated APCs in a ratio 1:0 or 1:1 in DMEM supplemented with nonessential amino acids, penicillin, streptomycin, 10% fetal calf serum (all from GIBCO), as well as α‐CD28 antibody (0.2 μg/mL; BD Biosciences) and α‐CD16/CD32 (1:200; BD Biosciences). Proliferation of CD3^+^CD8^+^CD44^+^‐positive primed T cells was evaluated via Cell Trace Violet attenuation assessed by flow cytometry.

### Cell depletion

WT, Langerin‐DTR, and CD11b‐DTR mice were intranasally infected with 250 PFU of PR8. At day 5 postinfection, mice were treated with 0.5 μg of DT by intraperitoneal inoculation. A second dose of DT was administrated at day 7 postinfection. Depletion efficiency was >80% when assessed 24 h post‐DT administration (Supporting Information Fig. 3).

## Statistical methods

The statistical analysis of differences in mean values was determined with a two‐tailed Student's *t*‐test. To determine differences between groups mean values, a one‐way ANOVA was performed followed by a Bonferroni's posttest. Values of *p*< 0.05 were considered statistically significant. Statistical analysis was performed with GraphPad Prism software.

## Conflict of interest

The authors declare no financial or commercial conflict of interest.

**Abbreviations**
DT: diphtheria toxin
mLN: mediastinal LN
moDCs: monocyte‐derived dendritic cells
NP: nucleoprotein
PA: acidic polymerase

## Supporting information

As a service to our authors and readers, this journal provides supporting information supplied by the authors. Such materials are peer reviewed and may be re‐organized for online delivery, but are not copy‐edited or typeset. Technical support issues arising from supporting information (other than missing files) should be addressed to the authors.

Peer review correspondence

**Supporting Information Figure 1**. Upregulation of Ly6C after inflammation defines respiratory CD11b^+^ moDCs. Identification of moDCs in lungs by flow cytometry. WT mice were infected intranasally with 250 PFU of PR8 virus. Animals were euthanized 4 days post‐infection (dpi) and lung subsets were analyzed by flow cytometry. MoDCs were gated as CD11b^+^ Ly6Chi CD209^+^ cells in the DC gate (CD11c+ MHC class II^+^SSClow).
**Supporting Information Figure 2**. Gating strategy indicating equal identification of moDCs via Ly6C or Mar‐1/CD64 discrimination. G1: Light scatter gating; G2: Singlets; G3: CD11c (+); G4: MHC class II high; G5: Siglec‐F negative; G6: CD11b(+) DCs. Overlay plots show backgating of CD64(+) Mar‐1(+) cells and CD11b(+) Ly6C(+) cells indicating population overlap.
**Supporting Information Figure 3**. Depletion efficiency assessed by flow cytometry in the lungs of Langerin‐DTR mice 24 h post‐DT treatment and in the blood of CD11b‐DTR mice 24 h post‐treatment.
**Supporting Information Figure 4**. Representative plots of CD8 T cell Tetramer NP^+^ in the lung of WT and CCR2‐/‐ mice during memoring response after PR8 secondary challenge.
