# Supplementary material for: Monocyte‐derived dendritic cells enhance protection against secondary influenza challenge by controlling the switch in CD8+ T‐cell immunodominance
Source: Eur J Immunol. 2016 Dec 9;47(2):345–52. doi: 10.1002/eji.201646523 (PMC5324604; doi:10.1002/eji.201646523)
Supplement: Supplementary file 2 — Supporting Information Figure 1. Upregulation of Ly6C after inflammation defines respiratory CD11b+ moDCs. Identification of moDCs in lungs by flow cytometry. WT mice were infected intranasally with 250 PFU of PR8 virus. Animals were euthanized 4 days post‐infection (dpi) and lung subsets were analyzed by flow cytometry. MoDCs were gated as CD11b+ Ly6Chi CD209+ cells in the DC gate (CD11c+ MHC class II+SSClow). Supporting Information Figure 2. Gating strategy indicating equal identification of moDCs via Ly6C or Mar‐1/CD64 discrimination. G1: Light scatter gating; G2: Singlets; G3: CD11c (+); G4: MHC class II high; G5: Siglec‐F negative; G6: CD11b(+) DCs. Overlay plots show backgating of CD64(+) Mar‐1(+) cells and CD11b(+) Ly6C(+) cells indicating population overlap. Supporting Information Figure 3. Depletion efficiency assessed by flow cytometry in the lungs of Langerin‐DTR mice 24 h post‐DT treatment and in the blood of CD11b‐DTR mice 24 h post‐treatment. Supporting Information Figure 4. Representative plots of CD8 T cell Tetramer NP+ in the lung of WT and CCR2‐/‐ mice during memoring response after PR8 secondary challenge. [file EJI-47-345-s002.pdf]

European Journal of Immunology

Supporting Information for DOI 10.1002/eji.201646523

Jazmina L. G. Cruz, José V. Pérez-Girón, Anja Lüdtke, Sergio Gómez-Medina, Paula Ruibal, Juliana Idoyaga and César Muñoz-Fontela

Monocyte-derived dendritic cells enhance protection against secondary influenza challenge by controlling the switch in CD8$^+$ T-cell immunodominance

© 2016 WILEY-VCH Verlag GmbH & Co. KGaA, Weinheim

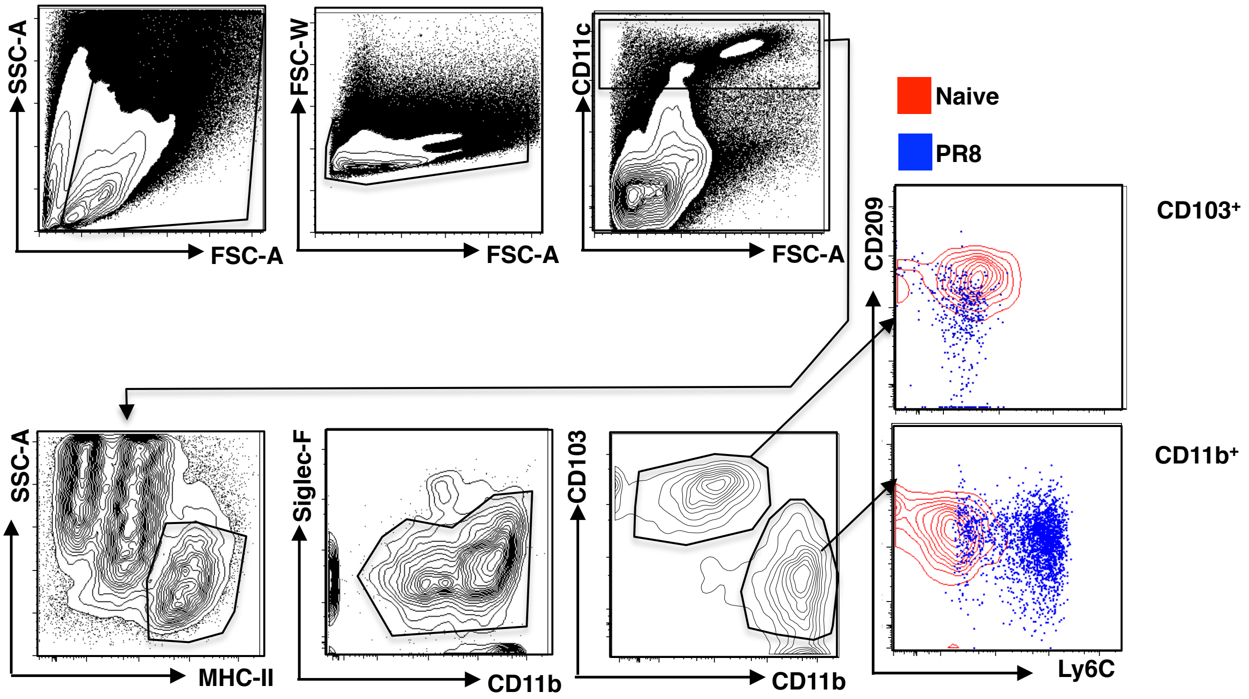

Supporting Information Figure 1. Upregulation of Ly6C after inflammation defines respiratory CD11b$^+$ moDCs. Identification of moDCs in lungs by flow cytometry. WT mice were infected intranasally with 250 PFU of PR8 virus. Animals were euthanized 4 days post-infection (dpi) and lung subsets were analyzed by flow cytometry. MoDCs were gated as CD11b$^+$ Ly6C^{hi} CD209$^+$ cells in the DC gate (CD11c$^+$ MHC class II$^+$ SSC^{low}).

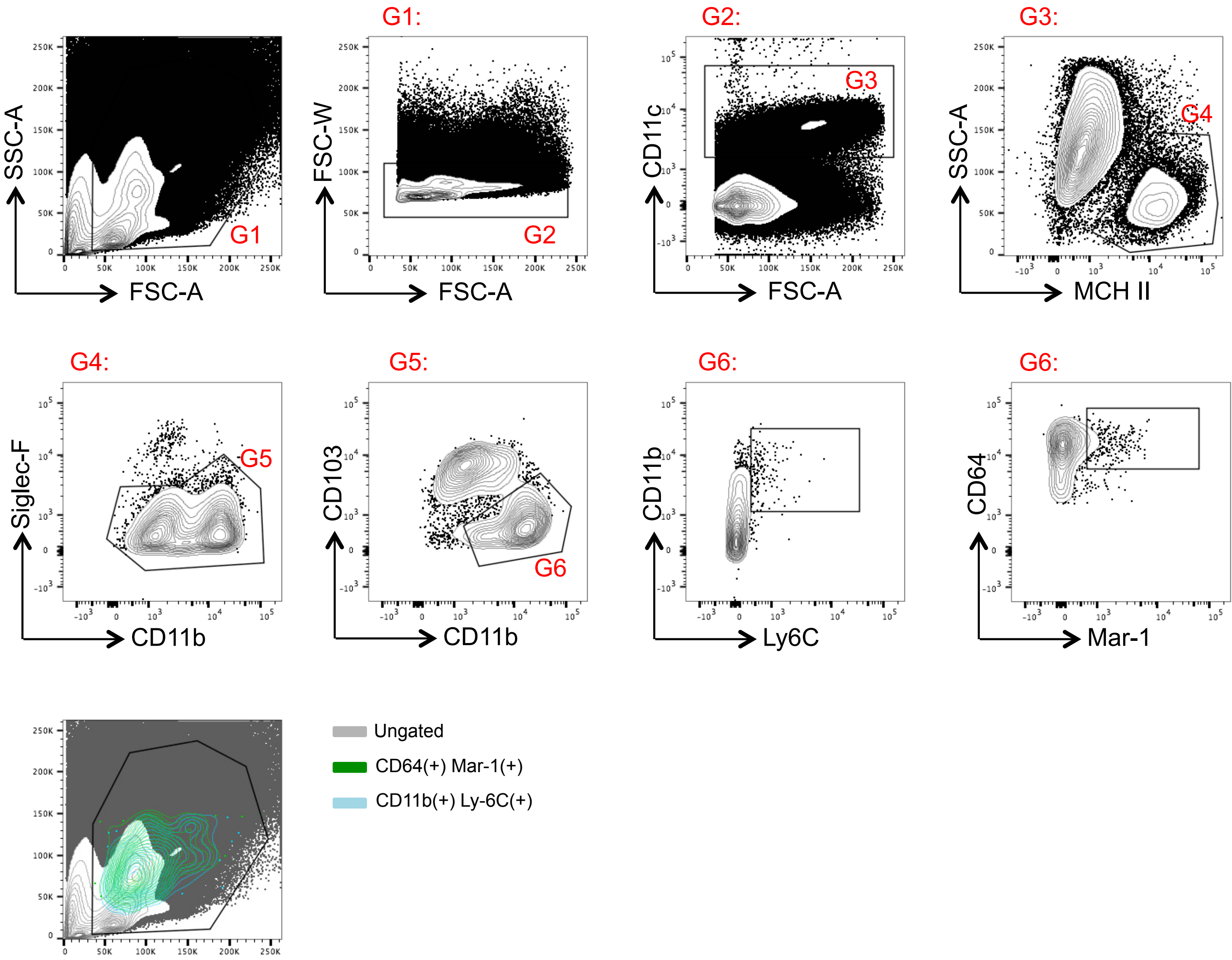

Supporting Information Figure 2. Gating strategy indicating equal identification of moDCs via Ly6C or Mar-1/CD64 discrimination. G1: Light scatter gating; G2: Singlets; G3: CD11c (+); G4: MHC class II high; G5: Siglec-F negative; G6: CD11b(+) DCs. Overlay plots show backgating of CD64(+) Mar-1(+) cells and CD11b(+) Ly6C(+) cells indicating population overlap.

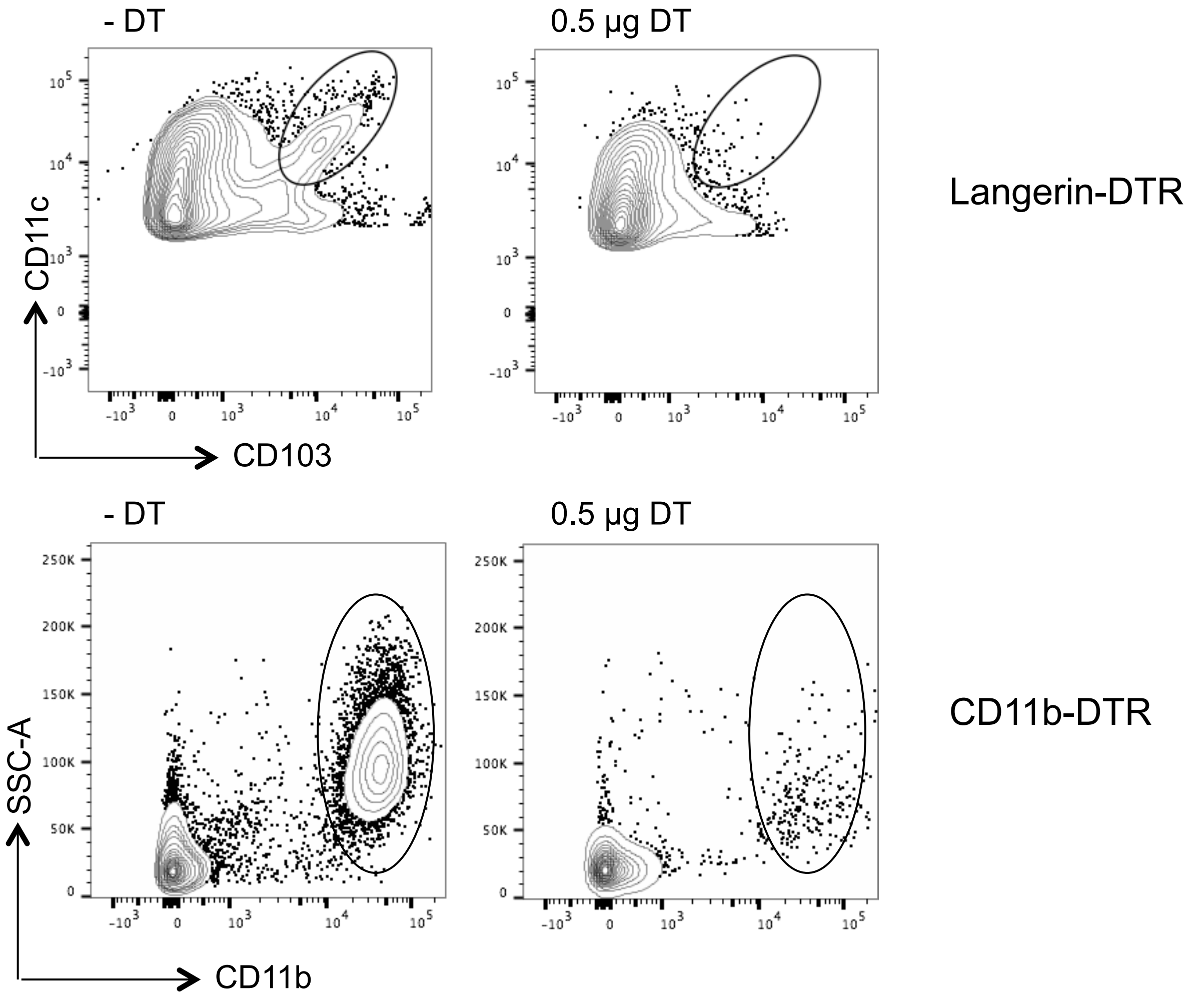

Supporting Information Figure 3. Depletion efficiency assessed by flow cytometry in the lungs of Langerin-DTR mice 24 h post-DT treatment and in the blood of CD11b-DTR mice 24 h post-treatment.

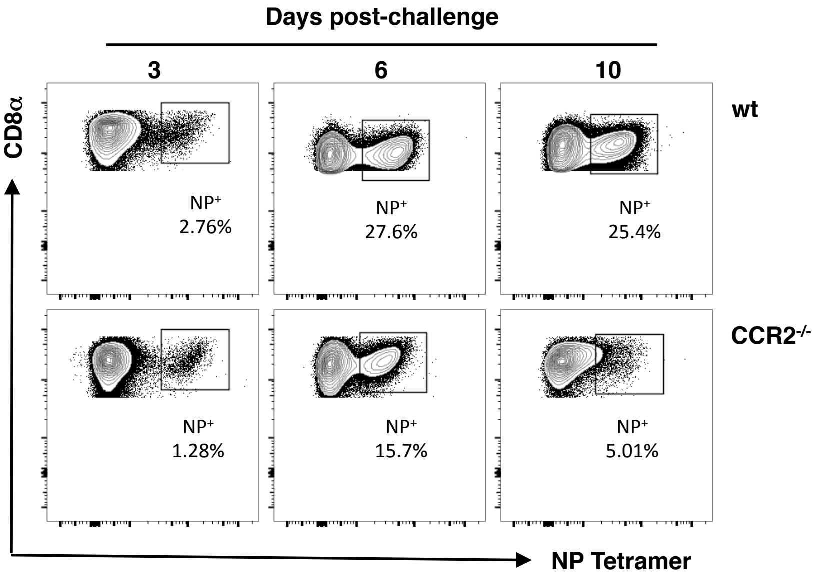

Supporting Information Figure 4. Representative plots of CD8 T cell Tetramer NP+ in the lung of WT and CCR2-/- mice during memoring response after PR8 secondary challenge.
