# Supplementary material for: Monocyte‐derived dendritic cells enhance protection against secondary influenza challenge by controlling the switch in CD8+ T‐cell immunodominance
Source: Eur J Immunol. 2016 Dec 9;47(2):345–52. doi: 10.1002/eji.201646523 (PMC5324604; doi:10.1002/eji.201646523)
Supplement: Supplementary file 1 — Peer review correspondence [file EJI-47-345-s001.pdf]

European Journal of Immunology

Peer review correspondence

Monocyte-derived dendritic cells enhance protection against secondary influenza challenge by controlling the switch in CD8+ T-cell immunodominance

Jazmina L.G. Cruz, José V. Pérez-Girón, Anja Lüdtke, Sergio Gómez-Medina, Paula Ruibal, Juliana Idoyaga and César Muñoz-Fontela

Corresponding author: César Muñoz-Fontela, Heinrich Pette Institute, Leibniz Institute For Experimental Virology, Hamburg, Germany

Review Timeline:	Submission date:	13 May 2016
	First editorial decision:	6 June 2016
	Revision received:	10 October 2016
	Accepted:	16 November 2016

Handling Executive Committee member: Prof. Annette Oxenius

Please note that the correspondence below does not include the standard editorial instructions regarding preparation and submission of revised manuscripts, only the scientific revisions requested and addressed.

First Editorial Decision – 6 June 2016

Dear Dr. Munoz-Fontela,

Manuscript ID eji.201646523 entitled "Monocyte-derived dendritic cells enhance protection against secondary influenza challenge through control of the CD8 T cell immunodominance switch" which you submitted to the European Journal of Immunology has been reviewed. The comments of the referees are included at the bottom of this letter.

A revised version of your manuscript that takes into account the comments of the referees will be reconsidered for publication. Should you disagree with any of the referees' concerns, you should address this in your point-by-point response and provide solid scientific reasons for why you will not make the requested changes.

You should also pay close attention to the editorial comments included below. *In particular, please edit your figure legends to follow Journal standards as outlined in the editorial comments. Failure to do this will result in delays in the re-review process.*

Please note that submitting a revision of your manuscript does not guarantee eventual acceptance, and that your revision will be re-reviewed by the referees before a decision is rendered.

If the revision of the paper is expected to take more than three months, please inform the editorial office. Revisions taking longer than six months may be assessed by new referee(s) to ensure the relevance and timeliness of the data.

Once again, thank you for submitting your manuscript to European Journal of Immunology and we look forward to receiving your revision.

Yours sincerely,
Karen Chu

On behalf of Prof. Annette Oxenius

Dr. Karen Chu
Editorial Office
European Journal of Immunology
e-mail: ejied@wiley.com
www.eji-journal.eu

****

Reviewer: 1

Comments to the Author
The paper by Cruz et al. is investigating the impact of monocyte-derived dendritic cells on induction of different antigen specific T cells, and their recruitment to the respiratory tract. The paper is elaborating on previous observations by others, some of which are sited in the paper, but the background is far from comprehensive enough to set the stage for this study.

Since much has been done in this field I think some of the relevant citations in this field are missing.
The authors have included the paper by Ballesteros-Tato et al. but others are missing such as:
1. J. Exp. Med. Volume 198, Number 3, August 4, 2003 399–410, by Crowe et al showing that antigen presenting cells preferentially drive NP specific T cells and
2. Eur J Immunol. 2011 September ; 41(9): 2632–2641 by Suárez-Ramírez et al that show CD103+ DCs are responsible for the recall response in CD8 T cells to influenza infection.

European Journal of Immunology

This would give a more differentiated picture of what has been done in the field before the paper.

With regard to the experiments in the paper I have some comments to the way data are presented and interpreted.

In fig 1D the authors have made adoptive transfer of bone marrow derived monocytes and 24 hours later isolated the transferred cells by gating strategy in flow cytometry to determine the CD11c and MHC-II expression. What are the numbers of cells that are isolated in this way? Since the histogram do not show any y-axis scaling it is difficult to see how many cells have in fact been analyzed. Secondly, and this is a general concern about all the flow cytometry data that are presented, there is not included any isotype-control, nor has it been stated in the M&M section if isotype-control has been used or how potential non-specific binding has been avoided in the paper. Surely the cells from PR8 infected mice represent an exudate with more inflammation going on, and since many monocytes and DCs unregulated Fc-receptors this must be controlled for. Especially the CD11c expression seem to be more limited in up regulation than MHC-II expression, and some of the cells from the infected animals have less expression of CD11c than the BM monocytes.

In Fig 2A I do not see the need for 4 lines in the plot. The group of animals that loses weight is a selected population, after the animals have been analyzed, and since the survival is shown in fig B it is not relevant to separate the animals within a group, rather show all animals from the same group as one.

In Fig 3 I have several comments to the experimental design and the way data is presented. Since this is a short communication without an extended interpretation/discussion by the authors it is important that the text and presented data are clearer to help the reader. Fig 3B+C are showing ratio rather than percentage or absolute numbers, this prevent the reader from seeing if numerator is increased, denominator decreased, or both, thus it is not possibly to interpret which of the two antigen specific subset that are affected by the treatment. Both B and C are lacking to show that the treatment has in fact worked, since no data or information in the text is provided to see the depletion of relevant cell subset, i.e. this is not a result of some non-specific effect in the animals.
In Fig 3D the authors have done proliferation assay with “responder T cells” from MLN, but what are these cells in fact representing. They are stated to be CD3+CD8+CD44high, but this includes both effector and memory cells, and the antigen specificity of these cells has not been determined. It would be better to isolate tetramer positive cells for the study, since this would eliminate any question if this is a non-specific proliferation that is demonstrated.
It is not stated in the experiment for how long the cell culture has incubated before analysis, but anyway if one look at the four plots in fig 3D there is a population (11% in the control) with very low violet tracer amount which indicated that some cells have undergone massive proliferation, surely that is not the case?

European Journal of Immunology

And since they are present in the control plot, lacking APCs, it must reside in the T cell population, but what are they?
In the three populations of APCs sorted in fig 3D it seems like moDCs are best at inducing proliferation, but several reasons for this could be suggested. Is it because only moDCs are presenting antigen taken up in situ or is because only moDCs have some proliferation inducing capacity that is lacking from the other two sets of APCs? As a minimum the authors should include the same APCs isolated by cell sorting and then pulse them with relevant peptides to demonstrate that the capacity to induce proliferation in fact is laying in the moDCs, alternative the sorted cells should also be analyzed for expression of relevant makers such as MHC-II, CD40 etc.

The data in the supplementary figure is overinterpreted. It is stated in the text P. 5 L. 14-17 that a population that is CD11b+ is found to be CD209+ and Ly6C+ but looking at the figure (population 2) there is no difference between cells from naïve and infected animals in terms of this expression, rather the population from infected animals have a tendency to a decreased expression of CD209. Since neither isotype control nor way of setting cut-off has been included the interpretation is resting on the authors' opinion, but it is not substantiated. A minor comment, the gating strategy includes a FSC-A vs. FSC-W plot, presumably to include singlet's. This assumption is only valid for round "lymphocyte-like" cells, has the authors and reason to believe the APC/DCs behave like lymphocytes in this manner?

Minor comments

In the text P. 8 L. 9 it says fig 3C, should be B.
In the text P. 8 L. 17 and P. 12 L. 2-3 it is stated that DT injection is done day 5, yet Fig 3C text P. 18 L. 20-21 says day 5 and 7 and the figure it self only indicate day 5.

Reviewer: 2

Comments to the Author
The study submitted by Cruz and colleagues examines the role of inflammatory moDCs (derived from circulating monocytes) in influenza A virus pathogenesis. This adds to an already substantial body of literature with a focus on the role these cells have in regulating T cell immuonodominance hierarchies. Overall the study is well done, with experiments and the results following a logical progression, and conclusions largely supported by the data.

The main issue is that the rationale for looking at the role of these cells was based on secondary responses to IAV challenge whereby CCR2-/- mice demonstrated increased pathology (as measured by weight loss) and delayed viral clearance, as compared to wildtype controls. The assumption presumably is

European Journal of Immunology

that there is a defect in the 2ry DbNP366 response which immunodominates the DbPA (and other CTL responses for that matter). To address this, the authors examine the ratio of DbNP:DbPA in a primary challenge, at a time whereby the peak DbNP366 response kicks in (day 9-10). The subsequent experiments show that it is the moDCs that are key in a decreased DbNP366 primary response.

What the authors fail to show is that this inability to sustain a primary DbNP366 response results in a defective memory response that correlates with a defect in viral clearance. As such, this last part of the study, which is the crux of their conclusion is lacking. The authors need to examine the DbNP366 response after secondary challenge in the CCR2-/- mice compared to WT controls. Moreover, it would be of interest to know if there was depletion of the CD11b+ cells during the secondary response, whether this would also impact the DbNP366 response. This would then determine whether a deficient DbNP366 response was due to defective priming (as asserted by the authors) or whether these same cells play a role in driving the DbNP366 response during a secondary challenge (and hence would also explain the observed results with regards to susceptibility).

First revision – authors’ response – 10 October 2016

Reviewer 1

1- Since much has been done in this field I think some of the relevant citations in this field are missing. The authors have included the paper by Ballesteros-Tato et al. but others are missing such as:
1. J. Exp. Med. Volume 198, Number 3, August 4, 2003 399–410, by Crowe et al showing that antigen presenting cells preferentially drive NP specific T cells and
2. Eur J Immunol. 2011 September ; 41(9): 2632–2641 by Suárez-Ramírez et al that show CD103+ DCs are responsible for the recall response in CD8 T cells to influenza infection.
This would give a more differentiated picture of what has been done in the field before the paper.

Agreed. We have introduced the suggested references in the Introduction section of the revised manuscript and discussed the goals of our study in the context of these previous findings (Lines 77-87).

2- In fig 1D the authors have made adoptive transfer of bone marrow derived monocytes and 24 hours later isolated the transferred cells by gating strategy in flow cytometry to determine the CD11c and MHC-II expression. What are the numbers of cells that are isolated in this way? Since the histogram do not show any y-axis scaling it is difficult to see how many cells have in fact been analyzed.

European Journal of Immunology

The frequency of recovered cells after adoptive transfer was 0.09 % of the Singlet population and 3% of the CD11c+ MHC class IIhi cell population. This information has been now added to the revised Fig. 1D and the revised Figure Legend 1 (Lines 447-448).

- Secondly, and this is a general concern about all the flow cytometry data that are presented, there is not included any isotype-control, nor has it been stated in the M&M section if isotype-control has been used or how potential non-specific binding has been avoided in the paper. Surely the cells from PR8 infected mice represent an exudate with more inflammation going on, and since many monocytes and DCs unregulated Fc-receptors this must be controlled for. Especially the CD11c expression seem to be more limited in up regulation than MHC-II expression, and some of the cells from the infected animals have less expression of CD11c than the BM monocytes.

Agreed. Specific information on flow panel design, isotype controls and measures to avoid non-specific binding has been added to the methods section of the manuscript (Lines 268-274).

3- In Fig 2A I do not see the need for 4 lines in the plot. The group of animals that loses weight is a selected population, after the animals have been analyzed, and since the survival is shown in fig B it is not relevant to separate the animals within a group, rather show all animals from the same group as one.

Agreed. We have modified this figure (new Figure 2A) as suggested in the revised version of the manuscript.

4-In Fig 3 I have several comments to the experimental design and the way data is presented. Since this is a short communication without an extended interpretation/discussion by the authors it is important that the text and presented data are clearer to help the reader.
- Fig 3B+C are showing ratio rather than percentage or absolute numbers, this prevent the reader from seeing if numerator is increased, denominator decreased, or both, thus it is not possibly to interpret which of the two antigen specific subset that are affected by the treatment.

Agreed. In the new Figure 3 A-E we have removed the ratios and the data is now presented as frequency of CD8 T cells specific for either PA or NP.

Both B and C are lacking to show that the treatment has in fact worked, since no data or information in the text is provided to see the depletion of relevant cell subset, i.e. this is not a result of some non-specific effect in the animals.

Agreed. Specific percentages of depletion of target cells in CD11b and Langerin DTR mouse models are now disclosed in the Methods section of the revised manuscript (Lines 297-298). Depletion efficiency in all

European Journal of Immunology

experiments was higher than 80% (see new Supplementary Figure 3). Antibody-based depletion experiments (Gr-1 and Ly6G antibodies, former Figure 3B) did not have associated depletion efficiency data and therefore have been removed from the manuscript.

- In Fig 3D the authors have done proliferation assay with “responder T cells” from MLN, but what are these cells in fact representing. They are stated to be CD3+CD8+CD44high, but this includes both effector and memory cells, and the antigen specificity of these cells has not been determined. It would be better to isolate tetramer positive cells for the study, since this would eliminate any question if this is a non-specific proliferation that is demonstrated.

We agree with the reviewer that the term ‘responder cells’ does not reflect the nature of the T cells utilized in our assay (Figure 3F in the revised version of the manuscript). We have in fact used antigen-experienced ‘primed T cells’ isolated from lung-draining mediastinal lymph nodes from mice infected intranasal with influenza virus. The ability of cell sorting-purified antigen presenting cells to re-stimulate primed T cells compared to non-stimulated control T cells was the goal of the study. This experimental strategy is widely accepted to test antigen-stimulation ex vivo (i. e. Ballesteros-Tato et al., Immunity 2014; Crowe et al., JEM 2003; Cho et al, JEM 2007).

- It is not stated in the experiment for how long the cell culture has incubated before analysis, but anyway if one look at the four plots in fig 3D there is a population (11% in the control) with very low violet tracer amount which indicated that some cells have undergone massive proliferation, surely that is not the case? And since they are present in the control plot, lacking APCs, it must reside in the T cell population, but what are they?

We apologize that there were no details about our culture conditions. The cells were co-cultured for 72 hrs. We have now added this information to the Methods section (Line 286).

We agree that some cells undergo homeostatic proliferation as described by the reviewer, a phenomenon described in the literature (Ge et al., PNAS 2002). However, this is observed in all of our experimental conditions. Thus, we are not denying the existence of this homeostatic proliferation, but looking for differences between the experimental groups.

- In the three populations of APCs sorted in fig 3D it seems like moDCs are best at inducing proliferation, but several reasons for this could be suggested. Is it because only moDCs are presenting antigen taken up in situ or is because only moDCs have some proliferation inducing capacity that is lacking from the other two sets of APCs?

European Journal of Immunology

This is an interesting question raised by the reviewer and in fact is the topic of ongoing research in our lab. We still need to determine whether moDCs migrate from the lung to the mLNs or rather whether they infiltrate mLNs from the blood (Nakano et al., Nat Immunol 2009) and receive antigens from other migratory DCs. This question while interesting is outside of the scope of our manuscript.

- As a minimum the authors should include the same APCs isolated by cell sorting and then pulse them with relevant peptides to demonstrate that the capacity to induce proliferation in fact is laying in the moDCs,

The experiments in Fig.3F are confirmatory of the effect we already observed in vivo, namely, that depletion of CD103+DCs do not influence late formation of influenza-specific CD8+ T cells. These experiments were done to confirm that moDCs have the capacity to present antigen.

- alternative the sorted cells should also be analyzed for expression of relevant makers such as MHC-II, CD40 etc.

Actually, the cells are gated based on the expression of MHCII, and this marker is used for the sorting of CD103+ and moDCs.

- The data in the supplementary figure is overinterpreted. It is stated in the text P. 5 L. 14-17 that a population that is CD11b+ is found to be CD209+ and Ly6C+ but looking at the figure (population 2) there is no difference between cells from naïve and infected animals in terms of this expression, rather the population from infected animals have a tendency to a decreased expression of CD209.

Agreed. We have revised our definition of moDCs as indicated in the new text (Lines 115-117; Line 139-141) and the Supplementary Figure 1. In addition, taking into account the reviewer’s suggestion we have also identified moDCs alternatively based on Mar-1 and CD64 staining as suggested recently by Plantinga and colleagues. These two gating strategies identified the same population as shown in the new Supplementary Figure 2.

Since neither isotype control nor way of setting cut-off has been included the interpretation is resting on the authors’ opinion, but it is not substantiated.

As explained above, we have set up all our gatings in either FMO or isotype controls. We have clarified this point in the revised version of the M&M.

European Journal of Immunology

- A minor comment, the gating strategy includes a FSC-A vs. FSC-W plot, presumably to include singlet's. This assumption is only valid for round "lymphocyte-like" cells, has the authors and reason to believe the APC/DCs behave like lymphocytes in this manner?

Our gates were designed, as explained above, with the use of isotype controls and always confirming populations by backgating. Using backgating we have confirmed that the monocyte population follows into the singlet's gate.

Minor comments

In the text P. 8 L. 9 it says fig 3C, should be B.
In the text P. 8 L. 17 and P. 12 L. 2-3 it is stated that DT injection is done day 5, yet Fig 3C text P. 18 L. 20-21 says day 5 and 7 and the figure it self only indicate day 5.

We thank the reviewer for the careful revision of our manuscript. All mistakes have been corrected in the revised manuscript.

Reviewer: 2

Comments to the Author
The study submitted by Cruz and colleagues examines the role of inflammatory moDCs (derived from circulating monocytes) in influenza A virus pathogenesis. This adds to an already substantial body of literature with a focus on the role these cells have in regulating T cell immuonodominance hierarchies. Overall the study is well done, with experiments and the results following a logical progression, and conclusions largely supported by the data.
The main issue is that the rationale for looking at the role of these cells was based on secondary responses to IAV challenge whereby CCR2-/- mice demonstrated increased pathology (as measured by weight loss) and delayed viral clearance, as compared to wildtype controls. The assumption presumably is that there is a defect in the 2ry DbNP366 response which immunodominates the DbPA (and other CTL responses for that matter). To address this, the authors examine the ratio of DbNP:DbPA in a primary challenge, at a time whereby the peak DbNP366 response kicks in (day 9-10). The subsequent experiments show that it is the moDCs that are key in a decreased DbNP366 primary response.

What the authors fail to show is that this inability to sustain a primary DbNP366 response results in a defective memory response that correlates with a defect in viral clearance. As such, this last part of the study, which is the crux of their conclusion is lacking.

European Journal of Immunology

- The authors need to examine the DbNP366 response after secondary challenge in the CCR2-/- mice compared to WT controls.

Agreed. In the revised version of our manuscript and, as suggested by the reviewer, we have compared the ability of wt and CCR2-/- mice to generate NP-specific CD8 T cells in mice re-challenged with influenza virus. We now show evidence that reduced infiltration of moDCs in CCR2-/- mice results in reduced formation of NP-specific CD8 T cells (new Figure 3D and E). Additionally, moDCs were necessary also for the generation of PA-specific CD8 T cells albeit at lower levels, which was consistent with previous findings (La Gruta et al., PNAS 2006; Crowe et al., JEM 2003).

- Moreover, it would be of interest to know if there was depletion of the CD11b+ cells during the secondary response, whether this would also impact the DbNP366 response.

Throughout the manuscript we present evidence that among CD11b(+) cells, moDCs are responsible for the late priming of influenza-specific CD8 T cells. Since the CD11b compartment is heterogeneous (Miller et al., Immunity 2012; Haniffa et al., Adv Immunol 2013) we decided to focus on the role of moDCs through the well-established CCR2ko model. This new data is discussed above.

Second Editorial Decision – 31 October 2016

Dear Dr. Munoz-Fontela,

It is a pleasure to provisionally accept your manuscript entitled "Monocyte-derived dendritic cells enhance protection against secondary influenza challenge through control of the CD8 T cell immunodominance switch" for publication in the European Journal of Immunology. For final acceptance, please follow the instructions below and return the requested items as soon as possible as we cannot process your manuscript further until all items listed below are dealt with.

Please note that EJI articles are now published online a few days after final acceptance (see Accepted Articles: http://onlinelibrary.wiley.com/journal/10.1002/(ISSN)1521-4141/accepted). The files used for the Accepted Articles are the final files and information supplied by you in Manuscript Central. You should therefore check that all the information (including author names) is correct as changes will NOT be permitted until the proofs stage.

We look forward to hearing from you and thank you for submitting your manuscript to the European Journal of Immunology.

Peer review correspondence

Yours sincerely,

Karen Chu

on behalf of Prof. Annette Oxenius

Dr. Karen Chu
Editorial Office
European Journal of Immunology
e-mail: ejied@wiley.com
www.eji-journal.eu
